# Supplementary material for: The Caspase Homologues in Scallop Chlamys farreri and Their Expression Responses to Toxic Dinoflagellates Exposure
Source: Toxins (Basel). 2022 Jan 31;14(2):108. doi: 10.3390/toxins14020108 (PMC8878197; doi:10.3390/toxins14020108)
Supplement: Supplementary file 1 [file toxins-14-00108-s001.zip › toxins-1573788 supplementary.pdf]

Supplementary Materials: The Caspase Homologues in Scallop *Chlamys farreri* and Their Expression Responses to Toxic Dinoflagellates Exposure

Zhongcheng Wei, Wei Ding, Moli Li, Jiaoxia Shi, Huizhen Wang, Yangrui Wang, Yubo Li, Yiqiang Xu, Jingjie Hu, Zhenmin Bao and Xiaoli Hu

Supplementary Results

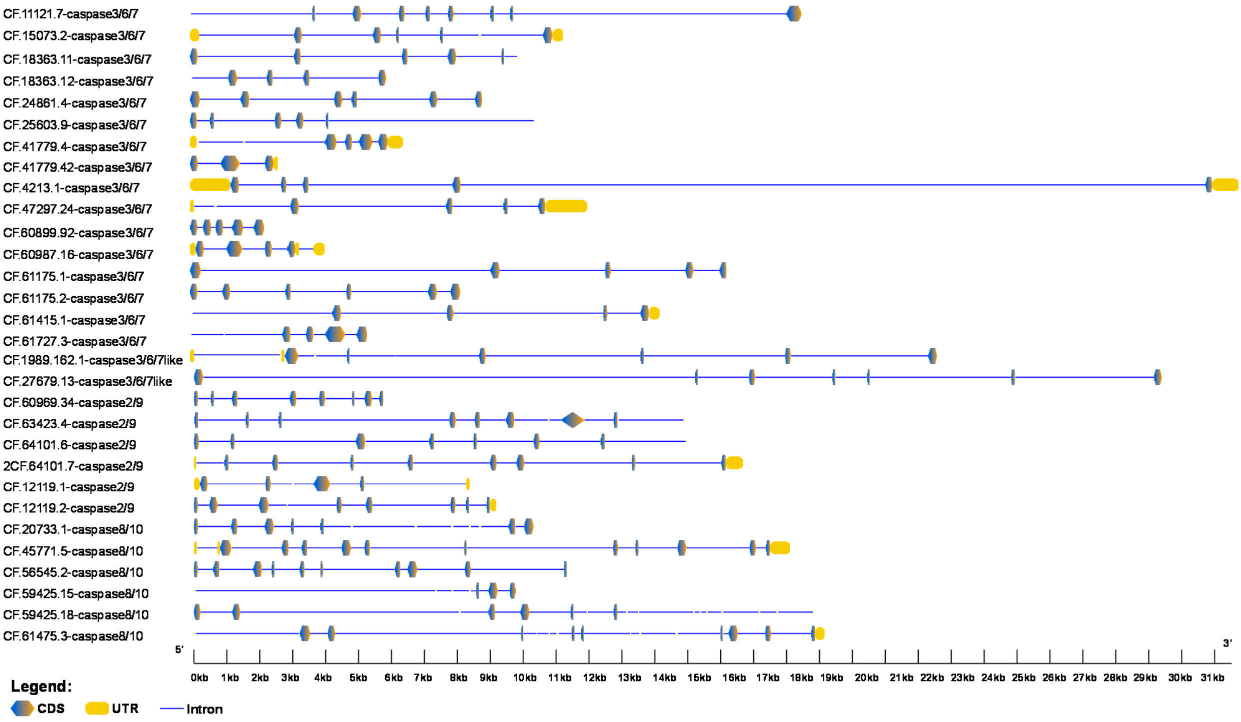

Figure S1. The structure of caspase homologues in *C. farreri*.

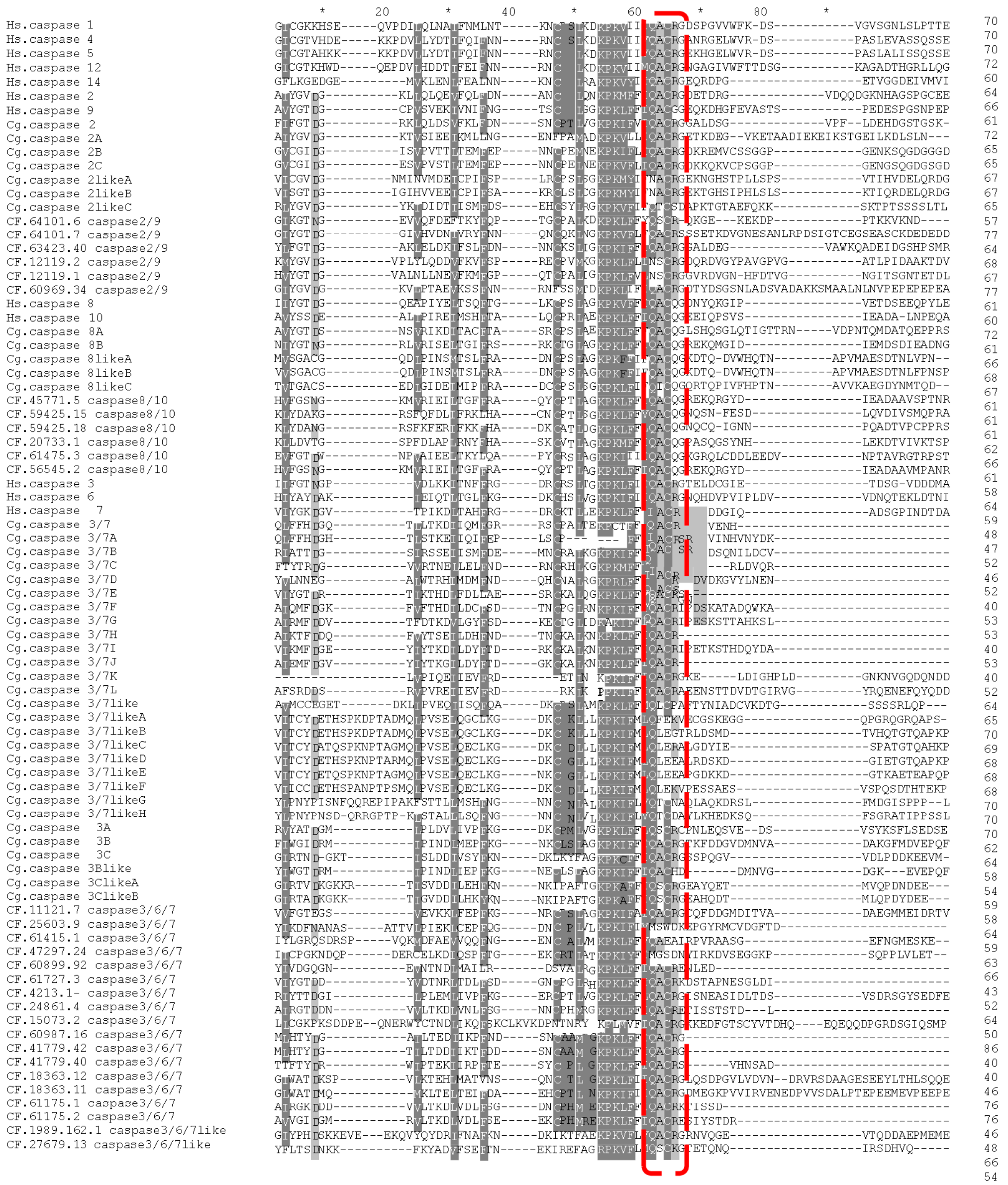

Figure S2. Alignment of part of the CASc domain sequences in *C. farreri.*
